# Author Correction: Wilkes subglacial basin ice sheet response to Southern Ocean warming during late Pleistocene interglacials

**DOI:** 10.1038/s41467-022-34002-4

**Published:** 2022-10-26

**Authors:** Ilaria Crotti, Aurélien Quiquet, Amaelle Landais, Barbara Stenni, David J. Wilson, Mirko Severi, Robert Mulvaney, Frank Wilhelms, Carlo Barbante, Massimo Frezzotti

**Affiliations:** 1 Department of Environmental Sciences, Informatics and Statistics, Ca’ Foscari University, Venice, Italy; 2 Laboratoire des Sciences du Climat et de l’Environnement LSCE/IPSL, CEA-CNRS-UVSQ, Université Paris-Saclay, Gif-sur-Yvette, France; 3 NumClim Solutions, Palaiseau, France; 4 Université Grenoble Alpes, CNRS, IRD, Grenoble INP, IGE, Grenoble, France; 5 Institute for Polar Sciences (ISP), CNR, Venice, Italy; 6 Institute of Earth and Planetary Sciences, University College London and Birkbeck, University of London, London, UK; 7 Department of Chemistry Ugo Schiff, University of Florence, Florence, Italy; 8 British Antarctic Survey, Cambridge, UK; 9 Alfred-Wegener-Institut Helmholtz-Zentrum für Polar-und Meeresforschung, Bremerhaven, Germany; 10 Geoscience Center, University of Göttingen, Göttingen, Germany; 11 Department of Science, Roma Tre University, Rome, Italy

**Keywords:** Palaeoclimate, Cryospheric science

Correction to: *Nature Communications* 10.1038/s41467-022-32847-3, published online 10 September 2022

The original version of this Article contained formatting errors in Table 1. The time interval was reported as 11–127 ka, this has been corrected to 117–127 ka. The column labels and data were incorrectly aligned. This has been corrected in both the PDF and HTML versions of the Article.

