# Supplementary material for: Wilkes subglacial basin ice sheet response to Southern Ocean warming during late Pleistocene interglacials
Source: Nat Commun. 2022 Sep 10;13:5328. doi: 10.1038/s41467-022-32847-3 (PMC9464198; doi:10.1038/s41467-022-32847-3)
Supplement: Supplementary file 1 — Supplementary Information [file 41467_2022_32847_MOESM1_ESM.pdf]

Wilkes Subglacial Basin Ice Sheet response to Southern Ocean Warming During Late Pleistocene Interglacials

Supplementary Information

Refined age model for core U1361A

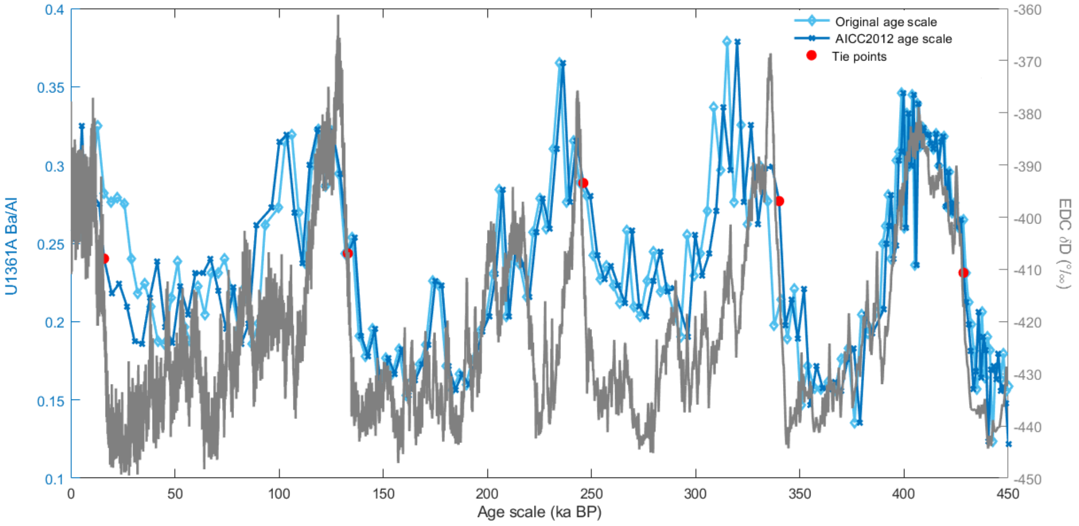

Supplementary Figure 1: Refined age model for the U1361A sediment core on AICC2012 age scale. The U1361A Ba/Al record is shown on its original age scale (light blue curve with diamonds[1]) and on the AICC2012 age scale (blue curve with crosses). The age scale transfer is performed through the alignment of the Ba/Al record with the EDC δD profile (grey curve) on the AICC2012 age scale[2]. Tie points are represented by red dots.

GRISLI ice sheet model – sensitivity tests

A total of 27 GRISLI simulations were carried out in order to evaluate the ice volume changes in the Wilkes Subglacial Basin and the ice thickness variations at Talos Dome during the past 400 ka. Because there are some slow feedbacks that affect the Antarctic ice sheet (e.g. glacial isostasy, internal temperature), the simulated state of the ice sheet for a given interglacial largely depends on the preceding glacial cycle. For this reason, we performed three different families of sensitivity tests in which we changed the initial state of the Antarctic ice sheet (glacial, GS; interglacial, IS; and deglaciated, DS) at 400 ka as described in the main text. The oceanic forcing is the main driver for glacial-interglacial grounding line migration. However, glacial-interglacial ocean temperature changes around Antarctica are poorly constrained, with no available record of sub-shelf temperatures in the SO spanning the past 400 ka, so this forcing represents an important source of uncertainty in the ice sheet evolution. For this reason, we performed GRISLI simulations prescribing three different oceanic forcing indexes: (i) derived from the North Atlantic ODP 980 benthic temperature record[3] (prescribed by Quiquet et al.[4]); (ii) derived from the stacked deep-sea benthic oxygen

isotope record (LR04)[5,6]; and (iii) derived from the EDC δD record[6,7] (Supplementary Figure 2). For both the LR04 and EDC indexes, we use a conversion factor so that the amplitude of the sub-shelf melt change from the Last Glacial Maximum to the present-day is similar to the original index (ODP 980), since the model was calibrated using this index[4]. We scaled the indexes so that sub-shelf melting is virtually suppressed during the Last Glacial Maximum (100% reduction but with a minimum melt rate of 1cm/year) but remains unchanged for the present-day. Given that the oceanic forcing represents a large source of uncertainty, we conducted model runs with three melting scenarios (standard, +5% and +10%) for each of the initial ice sheet conditions (glacial, interglacial, and deglaciated).

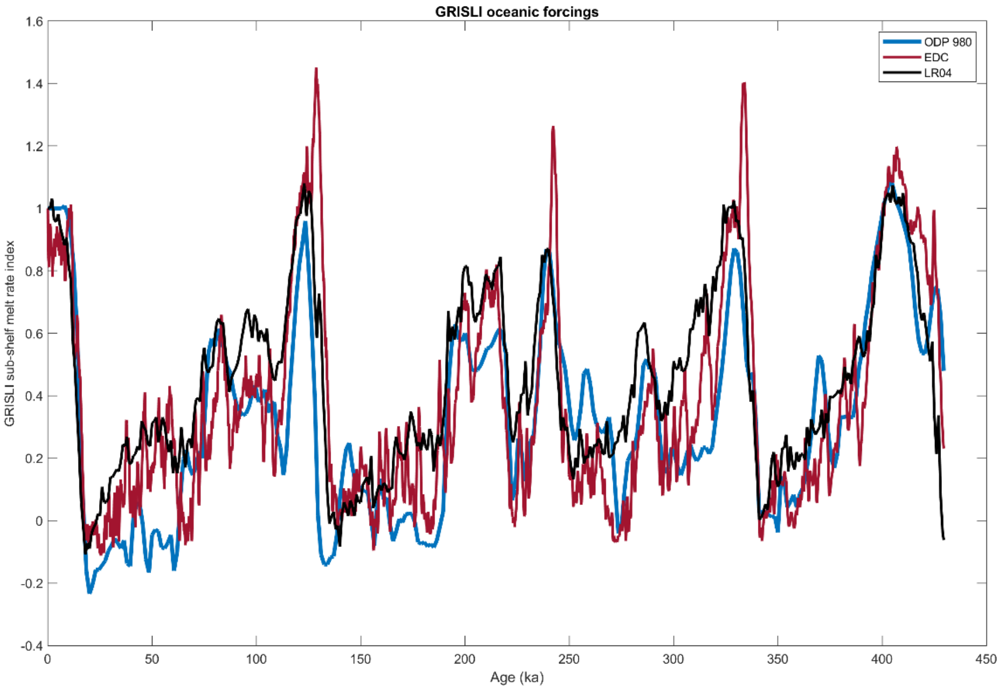

Supplementary Figure 2: Oceanic forcing indexes for the GRISLI simulations over the past 400 ka. The three indexes were derived from the ODP 980 bottom water temperatures[3] as applied in the original model simulation[4] (blue curve), the EDC δD profile[8] (dark red curve), and the LR04 benthic $\delta^{18}O$ stack[5] (black curve).

In order to assess the differences between the sensitivity tests forced by the NADW temperature record from ODP 980[4], the LR04 oxygen isotope stack[5], and the EDC record[8], we compare (i) the elevation changes at Talos Dome simulated with GRISLI over the past 350 ka (Supplementary Figure 3) and (ii) the simulated Wilkes Subglacial Basin ice volume changes over the past 350 ka (Supplementary Figure 4). The drainage basin boundaries used to calculate ice volume changes in the Wilkes Subglacial Basin are from IMBIE2, extended offshore for the ISMIP6 project[9]. Elevation variations at Talos Dome during each interglacial are calculated for all the simulations and shown in Supplementary Table 1.

The IS simulations, have been disregarded due to the collapse of the Wilkes Subglacial Basin ice sheet during MIS 9.3 for all the tests, which is inconsistent with the TALDICE record. In addition, the DS and GS simulations forced by the EDC and LR04 oceanic indexes produce as well a collapse of the Wilkes Subglacial Basin ice sheet during MIS 5.5 and MIS 9.3, and a strong ice thinning at Talos Dome and large ice volume decrease in the Wilkes Subglacial Basin, that is irreconcilable with our data. Therefore, we discuss only the GS and DS simulations forced by the North Atlantic oceanic record (Quiquet et al.[4] forcing) in the main text.

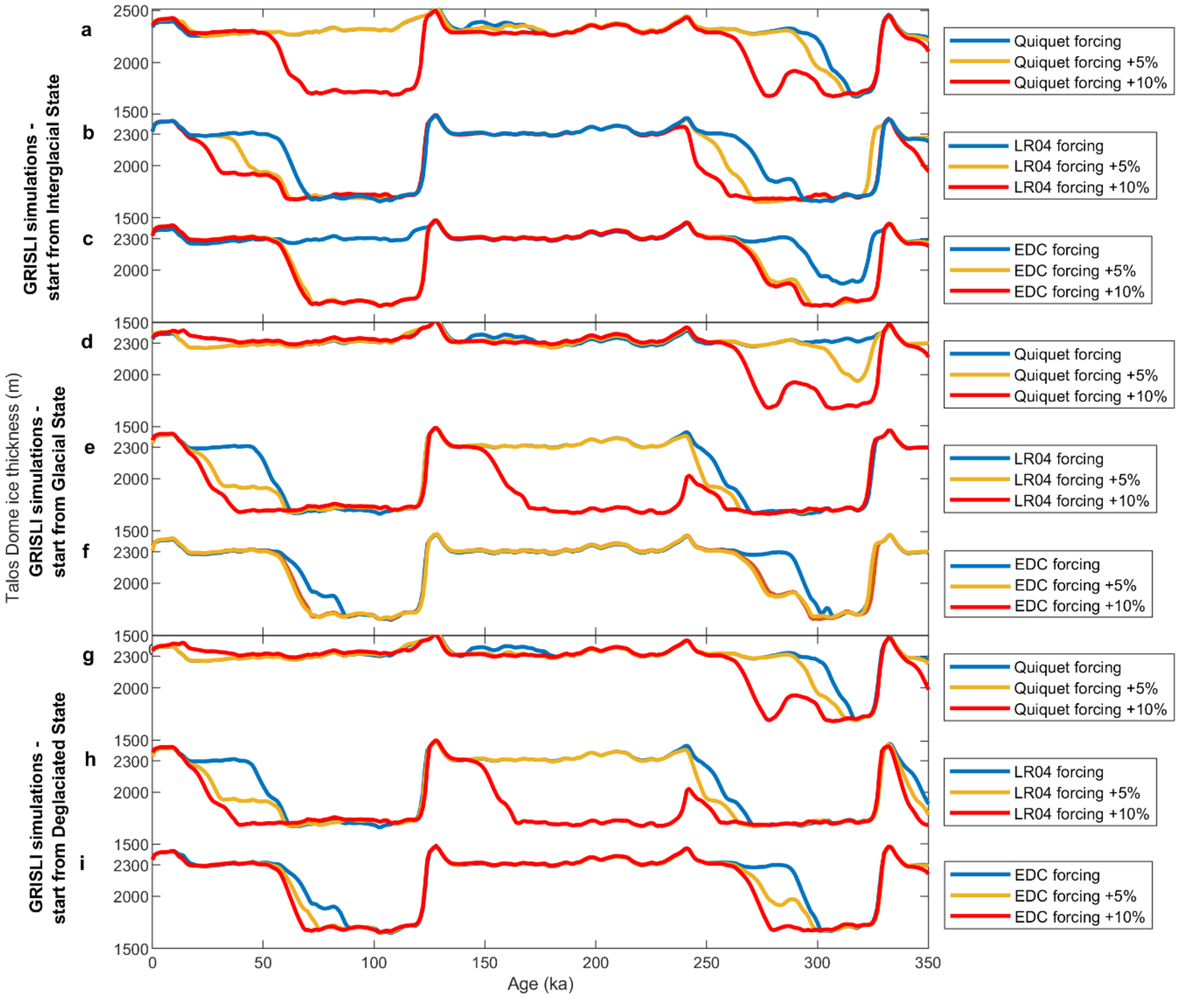

Supplementary Figure 3: Ice thickness variations at Talos Dome from (a-c) GRISLI Interglacial State, (d-f) Glacial State, and (g-i) Deglaciated State simulations over the past 350 ka. We applied the NADW oceanic forcing from Quiquet et al. (2018)[4], the oceanic forcing derived from the LR04 benthic stack[5], and the oceanic forcing derived from the EDC δD record[8]. The simulations are forced with the original oceanic indexes (blue curves) and with the forcing increased by 5% (yellow curves) and 10% (red curves).

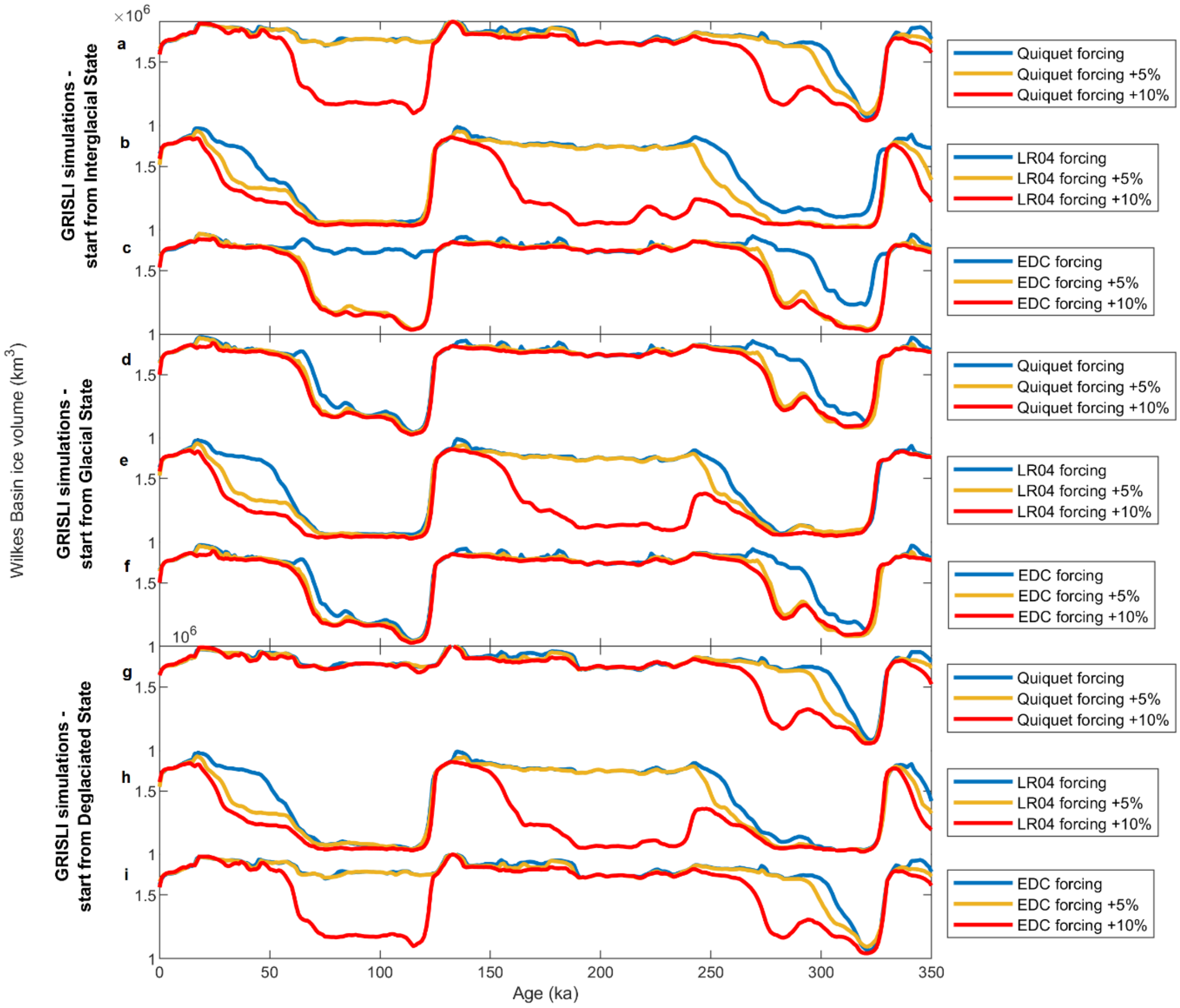

Supplementary Figure 4: Wilkes Subglacial Basin ice volume evolution from (a-c) GRISLI Interglacial State, (d-f) Glacial State, and (g-i). Deglaciated State simulations over the past 350 ka. We applied the NADW oceanic forcing from Quiquet et al. (2018)[4], the oceanic forcing derived from the LR04 benthic stack[5], and the oceanic forcing derived from the EDC δD record[8]. The simulations are forced with the original oceanic indexes (blue curves) and with the forcing increased by 5% (yellow curves) and 10% (red curves).

	Ocean forcing	GRISLI elevation changes at Talos Dome (m)		
Time interval for max elevation anomaly at TALDICE (ka)		115-128	233-241	321-332
IS	Quiquet et al. (2018)	-132	-103	-749
IS-5	Quiquet et al. (2018) +5%	-126	-101	-720
IS-10	Quiquet et al. (2018) +10%	-750	-87	-714
GS	Quiquet et al. (2018)	-126	-85	-134
GS-5	Quiquet et al. (2018) +5%	-116	-89	-473
GS-10	Quiquet et al. (2018) +10%	-152	-103	-754
DS	Quiquet et al. (2018)	-123	-103	-757
DS-5	Quiquet et al. (2018) +5%	-114	-104	-754
DS-10	Quiquet et al. (2018) +10%	-146	-105	-749
IS-EDC	EDC (Jouzel et al. 2007)	-167	-110	-487
IS-5-EDC	EDC +5%	-750	-109	-731
IS-10-EDC	EDC +10%	-757	-105	-731
GS-EDC	EDC	-746	-109	-703
GS-5-EDC	EDC +5%	-755	-107	-746
GS-10-EDC	EDC +10%	-757	-105	-722
DS-EDC	EDC	-758	-107	-742
DS-5-EDC	EDC +5%	-754	-108	-744
DS-10-EDC	EDC +10%	-760	-110	-751
IS-LR04	LR04 (Lisiecki & Raymo, 2005)	-755	-93	-709
IS-5-LR04	LR04 +5%	-769	-37	-738
IS-10-LR04	LR04 +10%	-758	+539	-721
GS-LR04	LR04	-771	-96	-735
GS-5-LR04	LR04 +5%	-765	-64	-712
GS-10- LR04	LR04 +10%	-760	-326	-714
DS-LR04	LR04	-766	-95	-736
DS-5-LR04	LR04 +5%	-766	-604	-737
DS-10-LR04	LR04 +10%	-765	+334	-723

Supplementary Table 1: Elevation changes modelled for Talos Dome with the GRISLI ice sheet model during interglacial MIS 5.5, 7.5, and 9.3. The elevation changes are modelled in 27 sensitivity tests, varying the Antarctic ice sheet initial conditions (IS, GS, DS) and the SO temperature forcing. Elevation variations are based on the interglacial time intervals when the simulated GRISLI ice thickness variations are maximized. The simulations are forced with the oceanic conditions derived from (i) Quiquet et al.[4], (ii) the EDC δD record[8], and (iii) the LR04 benthic stack[5].

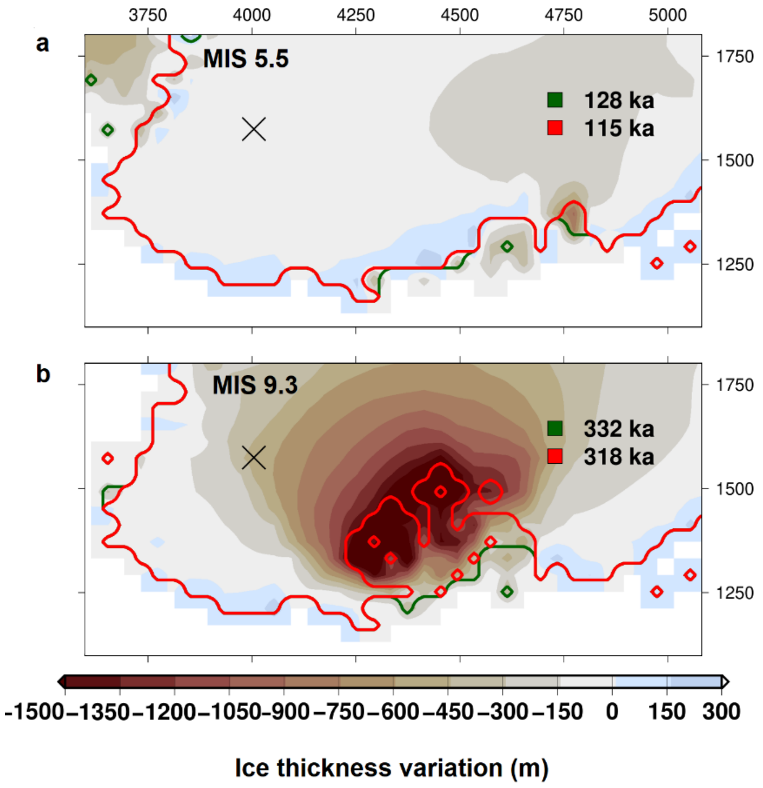

Supplementary Figure 5: Illustration of ice thickness variations in the vicinity of the Wilkes Subglacial Basin during (a) MIS 5.5 (115-128 ka), and (b) MIS 9.3 (318-332 ka), according to the GRISLI GS-5 simulation. The grounding line position at those different times is shown with green and red lines.

Supplementary references

1. Wilson, D. J. *et al.* Ice loss from the East Antarctic Ice Sheet during late Pleistocene interglacials. *Nature* **561**, 383–386 (2018).

2. Bazin, L. *et al.* An optimized multi-proxy, multi-site Antarctic ice and gas orbital chronology (AICC2012): 120-800 ka. *Clim. Past* **9**, 1715–1731 (2013).

3. Waelbroeck, C., Labeyrie, L., Michel, E., Duplessy, J. C. & Mcmanus, J. F. Sea-level and deep water temperature changes derived from benthic foraminifera isotopic records. *Quat. Sci. Rev.* **21**, 295–305 (2002).

4. Quiquet, A., Dumas, C., Ritz, C., Peyaud, V. & Roche, D. M. The GRISLI ice sheet model (version 2 . 0): calibration and validation for multi-millennial changes of the Antarctic ice sheet. *Geosci. Model Dev.* **11**, 5003–5025 (2018).

5. Lisiecki, L. E. & Raymo, M. E. A Pliocene-Pleistocene stack of 57 globally distributed benthic δ 18O records. *Paleoceanography* **20**, 1–17 (2005).

6. Golledge, N. R. *et al.* Antarctic contribution to meltwater pulse 1A from reduced Southern Ocean overturning. *Nat. Commun.* **5**, 1–10 (2014).

7. Blasco, J., Tabone, I., Alvarez-Solas, J., Robinson, A. & Montoya, M. The Antarctic Ice Sheet response to glacial millennial-scale variability. *Clim. Past* **15**, 121–133 (2019).

8. Jouzel, J. *et al.* Orbital and millennial antarctic climate variability over the past 800,000 years. *Science (80-.).* **317**, 793–796 (2007).

9. Nowicki, S. M. J. *et al.* Ice Sheet Model Intercomparison Project (ISMIP6) contribution to CMIP6. *Geosci. Model Dev.* **9**, 4521–4545 (2016).
